# Validating the Spanish translation of the posttraumatic stress disorder checklist (PCL-5) in a sample of individuals with traumatic brain injury

**DOI:** 10.3389/fpsyg.2024.1216435

**Published:** 2024-06-07

**Authors:** E. F. Haghish, Juan Sahuquillo, Andreea Radoi, Ingio Pomposo, Guillermo Carbayo Lozano

**Affiliations:** ^1^Department of Psychology, University of Oslo, Oslo, Norway; ^2^Neurovascular Research Laboratory, Vall d’Hebron Research Institute, Barcelona, Balearic Islands, Spain; ^3^BarcelonaBeta Brain Research Center, Barcelona, Catalonia, Spain; ^4^Department of Neurosurgery, Cruces University Hospital, Bilbao, Spain

**Keywords:** post-traumatic stress disorder, traumatic brain injury, PCL-5, Spanish, validation

## Abstract

**Introduction:**

There is controversy regarding the comorbidity of posttraumatic stress disorder (PTSD) and traumatic brain injury (TBI). The present study translated the PTSD Checklist for DSM-5 (PCL-5) to Spanish and validated it in a sample of patients with TBI 6 months after the injury.

**Methods:**

The study included 233 patients (162 males and 71 females) recruited from four Spanish hospitals within 24 h of traumatic brain injury. A total of 12.2% of the sample met the provisional PTSD diagnostic criteria, and the prevalence was equal between male and female participants.

**Results:**

The analysis confirmed the internal consistency of the translated instrument (*α* = 0.95). The concurrent validity of the instrument was confirmed based on high correlation coefficients of 0.7 and 0.74 with the General Anxiety Disorder-7 (GAD-7) and Patient Health Questionnaire (PHQ-9), respectively. Exploratory factor analysis also confirmed that the items on the PCL-5 can be differentiated from the GAD-7 and PHQ-9 items. Confirmatory factor analysis (CFA) was used to examine the structural validity of the Spanish translation of the PCL-5 with three different models. CFA partially confirmed the four-factor PTSD model, whereas both the six-factor anhedonia model and the seven-factor hybrid model showed adequate fit. However, the difference between the anhedonia and hybrid models was not statistically significant; moreover, both models showed signs of overfitting. Therefore, the utility of these models should be reexamined in future studies.

**Conclusion:**

Overall, the results suggest that the Spanish translation of the PCL-5 is a reliable and valid instrument for screening PTSD symptoms among Spanish TBI patients. The Spanish translation of the PCL-5 is also presented in the manuscript.

## Introduction

One of the widely used instruments for assessing posttraumatic stress disorder (PTSD) is the PTSD Checklist (PCL; Weathers et al., 1993; Blanchard et al., 1996), which was developed based on the DSM-IV diagnostic criteria for PTSD (American Psychiatric Association, 1994). The PCL has shown good psychometric properties for a variety of samples (Keen et al., 2008; McDonald and Calhoun, 2010). The fifth version of the DSM (DSM-5; American Psychiatric Association, 2013) introduces different classification criteria for PTSD, placing it in the category of trauma-and stressor-related disorders (American Psychiatric Association, 2013; Weathers et al., 2014); consequently, the PCL-5 questionnaire was developed to correspond to the new diagnostic criteria (Weathers et al., 2013). The PCL-5 has been validated for a variety of languages, such as Chinese, French, German, Persian, and Dutch (Wang et al., 2015; Ashbaugh et al., 2016; Bovin et al., 2016; Sadeghi et al., 2016; Krüger-Gottschalk et al., 2017; Van Praag et al., 2020). The current study is the first to translate this instrument to Spanish and examine the reliability and validity of the translated version in a sample of individuals with traumatic brain injury (TBI).

### PTSD models

There is controversy regarding the factorial structure of PTSD. The four-factor PTSD model derived from the symptom categories of the DSM-5 – frequently referred to as the DSM-5 PTSD model – is often criticized in the literature for lacking structural validity (Armour et al., 2015). The DSM-5 has eight criteria for the diagnosis of PTSD, and these criteria apply to adults, adolescents, and children older than 6 years (different PTSD criteria are provided for younger children). However, the main PTSD symptoms are divided in four categories: (B) intrusion, (C) avoidance, (D) negative alterations in cognitions and mood, and (E) alterations in arousal and reactivity. The other categories (A, F, G, and H) focus on additional diagnostic requirements, such as exposure to trauma (direct, indirect, witnessed, etc.) as well as the causes of the symptoms and the duration of the disturbance, which should last for a minimum of 1 month. Therefore, the four-factor model is constructed using the B, C, D, and E categories. In recent years, several PTSD models have been proposed to show a better fit than the aforementioned four-factor DSM-5 model. The majority of these models are consistent with DSM-5 symptoms and only vary in terms of suggesting different factors, breaking a category of symptoms into smaller groups of highly related symptoms. Two of these models have gained more popularity, i.e., the six-factor anhedonia model (Liu et al., 2014) and the seven-factor hybrid model (Armour et al., 2015). The anhedonia model breaks the “negative alterations in cognitions and mood” factor into two factors: “negative affect” and “anhedonia.” It also distinguishes “dysphoric arousal” from “anxious arousal” by dividing the “alterations in arousal and reactivity” factor. Like the anhedonia model, the hybrid model also suggested a division in the “negative alterations in cognitions and mood” factor. However, it suggests a three arousal factors instead of the “alterations in arousal and reactivity” factor, namely, “dysphoric arousal,” “anxious arousal,” and “externalizing behavior.”

### Comorbidity of PTSD and TBI

The prevalence of PTSD among individuals suffering from TBI has been an area of dispute (Jaffee et al., 2007). Recent studies suggest that PTSD occurs more frequently after mild TBI (Carlson et al., 2011; for a review, see Van Praag et al., 2019). The DSM-5 added a section for differential diagnosis between PTSD and TBI and warned that PTSD and TBI-related neurocognitive symptoms may overlap or occur concurrently. There are a few symptoms that are specific to PTSD, such as avoidance, as well as symptoms specific to TBI, such as disorientation and confusion. Nevertheless, there is a high risk of comorbidity between PTSD and TBI. For instance, the prevalence of PTSD is estimated to be as high as 32 to 66% among veterans with a history of TBI (Hoge et al., 2014), and for civilians with a history of TBI, it has been estimated to range from 11 to 40% across different reports (Bryant et al., 2001; Bombardier et al., 2006; Haarbauer-Krupa et al., 2017). For both veterans and civilians, the estimated occurrence rate has a wide range, which indicates that additional research on the prevalence of PTSD after TBI, as well as reliable PTSD screening instruments, is needed.

## Current study

The first aim of the current study was to translate the PCL-5 to the Spanish language. The second aim of the study was to assess the validity and reliability of the translated version among a sample of individuals with TBI. The third purpose of this study was to assess the prevalence of a provisional PTSD diagnosis among TBI patients 6 months after the injury. Finally, this study assessed three factorial models, including the four-factor PTSD model directly derived from the DSM-5, the anhedonia model and the hybrid model. Based on the literature, we expected that both models provide better fits than the four-factor model. To our knowledge, this manuscript is the first to publish a systematic translation of the PCL-5 to the Spanish language and examine its psychometric properties.

## Method

### Sample

The data used in this article are from 6-month postinjury assessments, as PTSD symptoms are expected to be more evident approximately 6 months after the TBI occurs (Bombardier et al., 2006; Haarbauer-Krupa et al., 2017). The data were obtained from four medical centers in Spain (Barcelona, Bilbao, Valencia, and Madrid) that participated in a prospective longitudinal nonrandomized observational study carried out by the Collaborative European NeuroTrauma Effectiveness Research in TBI (CENTER-TBI). The data for the CENTER-TBI study were collected through the *Quesgen* Electronic Case Report Form (e-CRF; Quesgen Systems, Inc., USA), hosted on the INCF platform^1^ and extracted via the INCF Neurobot tool (INCF, Sweden). The CENTER-TBI study included all participants who attended one of the involved hospitals within 24 h of the injury, had a clinical diagnosis of TBI with a clinical indication for CT scan, and signed an informed consent form to participate in the study. There was no age limit, and only patients with severe preexisting neurological disorders were excluded from the study. The current analysis, however, was carried out on participants who were at least 16 years old, which was the recommended age for the questionnaires used herein.

### Measures

The CENTER-TBI study included multiple outcome assessments conducted by a physician, neuropsychologist, and/or study nurse. The assessments were carried out 6 months after the TBI occurred. The data used herein only included the questionnaires completed by the patients 6 months after the injury. These instruments are further explained below.

#### PCL-5

Participants were asked to complete the 20-item PCL-5. Compared to the PCL, the most notable changes in the PCL-5 are the addition of three items for assessing the new PTSD symptoms recognized by the DSM-5: *blame*, *negative emotions* and *self-destructive behavior* (Blevins et al., 2015). Moreover, some of the items from the original PCL were reworded to reflect the new criteria of the DSM-5. Finally, the Likert rating scale was changed from 1–5 to 0–4. For example, the first item inquires “In the past month, how much were you bothered by repeated, disturbing, and unwanted memories of the stressful experience?,” and the possible responses range from 0 (not at all) to 4 (extremely). Therefore, the sum of the scores of the PCL-5 can range from 0 to 80. Even though the PCL-5 is a screening instrument and is not sufficient for making a diagnosis on its own, several cutoff values are suggested for provisional diagnosis, ranging from 28 to 38 (Ashbaugh et al., 2016). The commonly used cutoff score for the PCL-5 is 33, as recommended by Weathers et al. (2013).

The translation and linguistic validation of the PCL-5 from English to Spanish followed a strict procedure by a team of CENTER-TBI collaborators. First, two independent Spanish translations of the PCL-5 were made by native Spanish speakers who were fluent in English. Next, a consensus version was developed by combining the two translations. This version was further edited by a psychologist in collaboration with the translators to confirm the conceptual equivalence. Then, a native English speaker who was fluent in Spanish retranslated the consensus version, and all the translators involved in the procedure approved the back-translation to be compatible with the original version. Furthermore, a cognitive debriefing of the translated instrument was performed. Three PTSD patients and three healthy volunteers were interviewed and asked to comment on the items to ensure that participants comprehended the items as intended by the original instrument. Finally, after reviewing the results of the cognitive debriefing interviews, further adjustments were made to the translation by five language coordinators involved in the CENTER-TBI study. The Spanish translation of the PCL-5 is included in the Appendix.

#### PHQ-9

The PHQ-9 (Kroenke et al., 2001; Kroenke and Spitzer, 2002) is the mood subscale of the Patient Health Questionnaire (PHQ), which includes 9 items corresponding to depression criteria in the DSM-IV. The PHQ-9 items ask participants how often the symptoms have bothered them within the previous 2 weeks, and the responses are provided on a Likert scale ranging from 0 (not at all) to 3 (nearly every day). Therefore, the total score of the questionnaire ranges from 0 to 27. The PHQ-9 has been used for screening for depression (Kroenke et al., 2001; Kroenke and Spitzer, 2002; Löwe et al., 2004; Martin et al., 2006) and has been shown to exhibit good validity and reliability among the general population and TBI patients (Teymoori et al., 2020a,b).

#### GAD-7

The GAD-7 (Spitzer et al., 2006) is a brief, 7-item self-report scale for measuring the severity of general anxiety disorder (GAD). The questionnaire is based on the DSM-IV and assesses how often the subject has been affected by GAD symptoms within the last 2 weeks. Each item is answered on a Likert scale ranging from 0 (not at all) to 3 (nearly every day). The scale’s score is computed by summing the scores of the items, with the total score ranging from 0 to 21. The reliability and validity of the GAD-7 have been confirmed in different populations (Swinson, 2006; Löwe et al., 2008; Ruiz et al., 2011; Beard and Björgvinsson, 2014).

### Sociodemographic and health data

The sociodemographic background assessment included age, sex, race, level of education, employment status and marital status. Furthermore, participants were asked to provide details about the cause of their injury and their mental health history before the head injury. In particular, the participants were asked whether they had (1) sought treatment for problems related to use of alcohol or other substances, (2) sought treatment for mood or anxiety-related disorders (e.g., depression), (3) sought treatment for any other mental health problem, or (4) been admitted to the hospital for psychiatric reasons. A binary variable was created to mark preexisting mental health issues based on participants’ self-reported history of mental health, where 0 was lack of seeking help for mental health problems and 1 meant seeking help for one or more of the aforementioned categories. Finally, we also included patient type of admission to the hospital, which was categorized into three types: emergency (ER), admission (AD), and intensive care unit (ICU).

### Statistical analyses

#### Reliability

The reliability of the Spanish PCL-5 was examined at the item level and the scale level. At the item level, the items’ mean score and distribution skewness were evaluated to check for anomalies. At the scale level, Cronbach’s alpha, split-half, item-total correlations, and McDonald’s omega are reported. As a rule of thumb, the literature suggests that Cronbach’s alpha coefficients ranging from 0.7 to 0.95 indicate acceptable reliability (Tavakol and Dennick, 2011) at the group level. However, some studies have suggested that a minimum alpha of 0.90 is adequate and that an alpha of 0.95 is desirable when examining the clinical application of instruments at the individual level (Bland and Altman, 1997). For the split-half analysis, we used the R package psych version 1.7.8 (Revelle, 2017) with its default setting (10,000 random split-halves).

#### Validity

The concurrent validity of the Spanish PCL-5 was examined by evaluating the relationship of PTSD with depression and general anxiety measured at 6 months postinjury. PTSD was expected to be strongly correlated with depression and general anxiety. Additionally, the structural validity of the instrument was examined using both exploratory factor analysis (EFA) and confirmatory factor analysis (CFA). For the EFA, parallel analysis with the MinRes algorithm from the psych R package (Revelle, 2017) was carried out to identify the number of factors for the PCL-5 instrument. An additional parallel analysis and EFA were carried out to examine whether items in the PCL-5 cluster were affected by distinct factors and thus could be differentiated from items in the PHQ-9 and GAD-7 instruments. For the CFA, the four-factor DSM-5 model, the six-factor anhedonia (Liu et al., 2014) and the seven-factor hybrid models (Armour et al., 2015) were assessed. To carry out the CFA analyses, each item was specified to load on only one latent factor, and only correlations between the latent variables were allowed. The CFA analyses were performed using the weighted least square mean and variance adjusted (WLSMV) estimator (Muthén et al., 1997) in R statistical software version 3.4.1 and the lavaan package version 0.6.1 (Rosseel, 2012). The WLSMV estimator is known to be more appropriate than the maximum likelihood (ML) estimator for performing CFA analysis on ordinal variables (Beauducel and Herzberg, 2006) and can result in less biased estimations (Nussbeck et al., 2006). The fitness of the CFA models was evaluated with the chi-square goodness of fit, root mean square error of approximation (RMSEA), RMSEA confidence interval, standardized root mean square residual (SRMR), comparative fit index (CFI), and Tucker Lewis index (TLI). For evaluating the CFA results, values of CFI and TLI above 0.95, RMSEA less than 0.06, and SRMR below 0.08 were considered to indicate adequate model fit (Hu and Bentler, 1999). Despite the dominant role of goodness-of-fit chi-square statistics in model testing, the chi-square test is known to be too liberal (Bryant and Satorra, 2012). Furthermore, many factors can lead to a type I error, such as sample size, model complexity, multivariate normality, and skewness (Marsh et al., 1988; Green et al., 1997; Alavi et al., 2020), even when using the WLSMV estimator (Muthén et al., 1997). Therefore, we placed more weight on the other goodness-of-fit indices when interpreting the CFA results.

#### Explorative analysis

In addition to examining the reliability and validity of the instrument, we explored the relationships between demographic variables (gender, age, level of education, and prior mental health) and patient type of admission and the PTSD score using Pearson’s correlation analysis and linear regression in R statistical software.

## Results

### Descriptive analysis

Of the 392 individuals (269 men and 123 women) who participated in the CENTER-TBI study from Spain, 257 (65.6%) responded to the 6-month postinjury re-examination, and 135 participants (90 men and 45 women) did not return the questionnaires. Four participants (1.5%) completed less than 70% of the items on the PCL-5 and were therefore removed from the analysis. Of the remaining 253 participants, 175 were men (mean age = 43.7, SD = 16.66) and 78 were women (mean age = 54.58, SD = 23.05). Approximately 84% of the participants reported being white European. This sample size was consistent with the recommendations for CFA (e.g., above 200 and for each model variable more than 10 observations are available; see Alavi et al., 2020) and was considered to be large enough for the WLSMV estimator to provide stable and reliable parameter estimations (Nussbeck et al., 2006). The variables describing the socioeconomic background of the study sample are summarized in Table 1.

**Table 1:** Sociodemographic background of the study sample.

Characteristics	Number	Percentage
Gender		
*Men*	175	69.2%
*Women*	78	30.8%
Age		
*16–30*	59	23.3%
*31–45*	76	30.0%
*46–60*	53	21.0%
*>60*	65	25.7%
Education level		
*Nonresponse*	3	1.1%
*Up to high school*	189	74.7%
*Technical training*	11	4.4%
*College/University*	50	19.8%
Type of admission		
*Emergency (ER)*	70	27.7%
*Admission (AD)*	53	20.9%
*Intensive Care Unit (ICU)*	130	51.4%
Prior mental health problems		
*Nonresponse*	9	3.6%
*Yes*	63	24.9%
*No*	181	71.5%

The PCL-5 total scores (sum of items) were low and right-skewed (skew = 1.41), and the scores ranged from 0 to 72 (out of 80). As shown in Table 2, the mean PCL-5 score was equal for both men and women, although men scored higher on the *Intrusion* and *Avoidance* categories, and women scored higher on the *Cognitions & Mood* and *Arousal & Reactivity* categories. Females had significantly greater scores on the GAD-7 and PHQ-9 than males (GAD-7: t=2.73,p=0.007 ; PHQ-9: t=2.39,p=0.018 ).

**Table 2:** Mean and SD of the measured instruments based on sex.

	**Mean**			**Standard deviation**		
	**Male**	**Female**	**Total**	**Male**	**Female**	**Total**
PCL-5 (total)	14.09	14.09	14.09	16.01	12.75	15.05
Intrusion*	3.37	2.91	3.23	4.5	3.51	4.22
Avoidance*	1.43	1.09	1.32	2.07	1.56	1.93
Cognitions and Mood*	5.01	5.45	5.15	6.02	6.13	6.04
Arousal and Reactivity*	4.28	4.64	4.39	5.02	4.16	4.77
GAD-7	4.29	6.08	4.83	4.8	4.77	4.85
PHQ-9	5.07	6.91	5.63	5.83	5.46	5.77

*The maximum sum scores of the Intrusion (5 items), Avoidance (2 items), Cognitions/Mood (7 items), and Arousal (6 items) subscales are 20, 8, 28, and 24, respectively.

Using a cutoff value of 33 for the total score, 31 TBI patients (12.2%) met the criteria for a provisional PTSD diagnosis. The characteristics (i.e., distribution of sex, age, level of education, and type of admission) of these 31 participants were fairly comparable to those of the overall sample. However, nearly 39% of them reported prior mental health problems. Overall, the total score was marginally correlated with the demographic variables. For example, the PCL-5 score was negatively correlated with age ( r=−0.14,p=0.018 ) and positively correlated with a prior history of mental health problems ( r=0.13,p=0.036 ). However, the correlation between education level and PTSD was nonsignificant. Multiple linear regression analysis was used to examine the relationships of demographic variables (sex, age, years of education, prior history of psychological problems, and admission to the ER, AD or ICU) with the PTSD score. The results of the regression indicated that although the model was significant, it only explained 7% of the variance in PTSD scores ( $R^{2}=0.07,\;F\left(5,196\right)=4.02,\;p<0.001$ ). Prior mental health problems ( $\beta =7.18,\;\;t\left(3.02\right),\;p=0.003$ ) and admission to the ICU ( $\beta =5.68,\;\;t\left(2.67\right),p=0.008$ ) were the only significant factors.

### Internal consistency and reliability

The Cronbach’s alpha and McDonald’s omega coefficients were 0.94 and 0.95, respectively, indicating that the Spanish translation of the PCL-5 showed excellent internal consistency. Similarly, all the DSM-5 categories had good Cronbach’s alpha coefficients (intrusion = 0.88, avoidance = 0.82, negative alterations in cognitions and mood = 0.87, and alterations in arousal and reactivity = 0.84). Furthermore, using the split-half method, a reliability of 0.97 was computed for the instrument. Additional item-level information is presented in Table 3. All the items showed a reasonable correlation with the total score, and their low mean and positive skewness were in line with the low mean and positive skewness of the total score on the PCL-5.

**Table 3:** Item descriptive statistics for the Spanish version of the PCL-5, divided by DSM-5 categories.

		Mean	SD	Item-total cor.	Skewness	Kurtosis
Intrusive	1. Intrusive distressing memories	0.83	1.11	0.70	1.13	0.11
	2. Recurrent distressing dreams	0.42	0.85	0.67	2.09	3.48
	3. Flashbacks	0.57	0.96	0.70	1.70	2.07
	4. Psychological reactivity to cues	0.87	1.17	0.74	1.15	0.18
	5. Physiological reactivity to cues	0.55	0.98	0.81	1.79	2.29
Avoidance	6. Avoiding thoughts, memories, ..	0.72	1.05	0.71	1.42	1.10
	7. Avoiding external reminders	0.61	1.05	0.70	1.65	1.63
Cognitions and mood	8. Dissociative amnesia	1.22	1.52	0.52	0.77	−1.02
	9. Negative beliefs or expectations	0.51	0.94	0.66	2.03	3.70
	10. Distorted blame	0.67	1.06	0.68	1.51	1.35
	11. Persistent shame, guilt, fear, etc.	0.65	1.10	0.81	1.62	1.58
	12. Disengagement and apathy	0.78	1.15	0.73	1.33	0.61
	13. Feeling of detachment	0.68	1.10	0.75	1.50	1.03
	14. Lack of positive emotions	0.63	1.11	0.78	1.61	1.38
Arousal and reactivity	15. Irritability and anger outbursts	0.63	1.03	0.69	1.61	1.70
	16. Recklessness and self-destruction	0.31	0.70	0.49	2.59	6.81
	17. Hypervigilance	0.94	1.14	0.68	1.03	0.10
	18. Exaggerated startle response	0.73	1.07	0.73	1.18	0.13
	19. Concentration problems	0.87	1.16	0.74	1.18	0.30
	20. Sleep disturbance	0.91	1.23	0.71	1.19	0.24

#### Validity

The Pearson correlations of GAD-7 and PHQ-9 scores with PCL-5 scores were 0.68 ( p<0.001 ) and 0.69 ( p<0.001 ), respectively. As expected, there was a high positive correlation of 0.76 ( p<0.001 ) between the PHQ-9 and the GAD-7 total scores. To examine the structural validity of the Spanish translation of the PCL-5, parallel analysis was performed, which suggested two factors. The first factor included items in the Intrusive and Avoidance categories, and the second factor included items in the negative cognitive and mood category and the arousal and reactivity category. The only exceptions were the items assessing “hypervigilance,” which was loaded on the first factor instead of the second, as well as the items assessing “exaggerated startle response” and “persistent shame, guilt, and fear,” which had high loadings on both factors. EFA was subsequently used to examine whether the PCL-5, GAD-7, and PHQ-9 load on distinct factors, indicating that these instruments measure differentiable outcomes despite their strong correlation, comorbidity, and conceptual overlap. Parallel analysis suggested a four-factor solution, which is presented in Table 4. Overall, the items of each instrument grouped with one another, indicating that they are indeed differentiable.

**Table 4:** Factorial structure of the PTSD, GAD-7, and PHQ-9 items.

	Intrusive and avoidance	Cognition, mood, arousal, and reactivity	PHQ-9	GAD-7
* Flashbacks	0.93			
* Intrusive distressing memories	0.88			
* Avoiding external reminders	0.83			
* Avoiding thoughts, memories, …	0.81			
* Recurrent distressing dreams	0.71			
* Physiological reactivity to cues	0.71			
* Psychological reactivity to cues	0.67			
* Hypervigilance	0.57			
* Exaggerated startle response	0.47			
* Persistent shame, guilt, fear, etc.	0.58	0.34		
* Distorted blame	0.34	0.44		
* Feeling of detachment		0.8		
* Lack of positive emotions		0.8		
* Disengagement and apathy		0.7		
* Negative beliefs or expectations		0.42		
* Dissociative amnesia		0.41		
* Recklessness & self-destruction		0.37		
* Concentration problems		0.45	0.37	
* Irritability and anger outbursts				0.57
* Sleep disturbance				0.43
– Feeling down, depressed, or hopeless			0.76	
– Little interest or pleasure in doing things			0.75	
– Feeling tired or having little energy			0.66	
– Trouble concentrating on things			0.61	
– Moving or speaking so slowly			0.59	
– Thoughts that you would be better off dead			0.53	
– Feeling bad about yourself			0.45	
– Trouble falling or staying asleep, or sleeping too much				0.48
– Poor appetite or overeating				0.30
+ Feeling afraid, as if something awful might happen			0.36	
+ Not being able to stop or control worrying			0.35	0.58
+ Trouble relaxing				0.79
+ Becoming easily annoyed or irritable		0.34		0.79
+ Being so restless that it is hard to sit still				0.66
+ Worrying too much about different things				0.64
+ Feeling nervous, anxious, or on edge				0.58

The PCL-5 items are marked with an asterisk, the PHQ-9 items are marked with a minus sign, and the GAD-7 items are marked with a plus sign.

**Figure 1:**
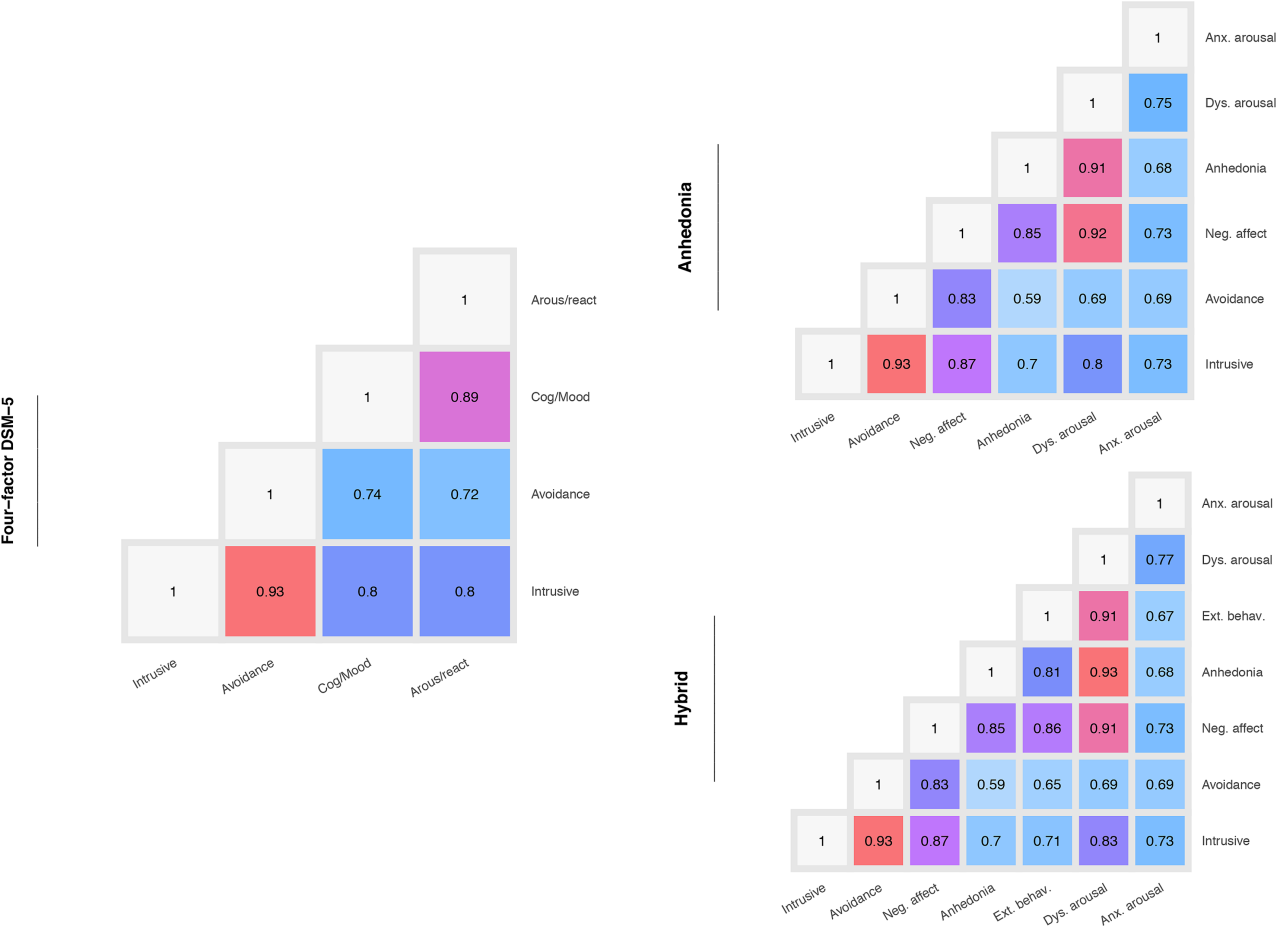
Correlations between factors of the analyzed models.

In addition, three confirmatory factor analyses were carried out, examining the four-factor DSM-5 model, the six-factor anhedonia model and the seven-factor hybrid model. Figure 1 shows the correlation between the factors of these models. As shown in this figure, in all the models, the factors had high correlation coefficients, ranging from 0.59 (between anhedonia and avoidance) to 0.93 (between avoidance and intrusive). However, the anhedonia and hybrid models seemed to have multiple factors with high correlations near 0.9 or above, which indicated that these factors might be redundant and could result in model overfitting.

**Figure 2:**
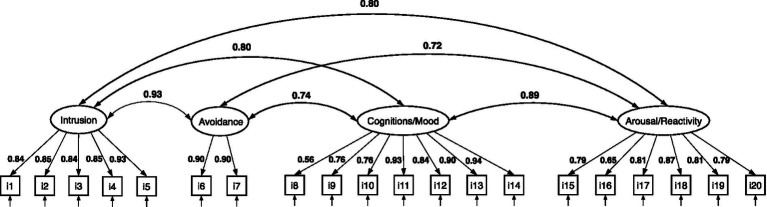
CFA of the four-factor PTSD model based on DSM-5 symptomatology. All estimates are standardized.

**Figure 3:**
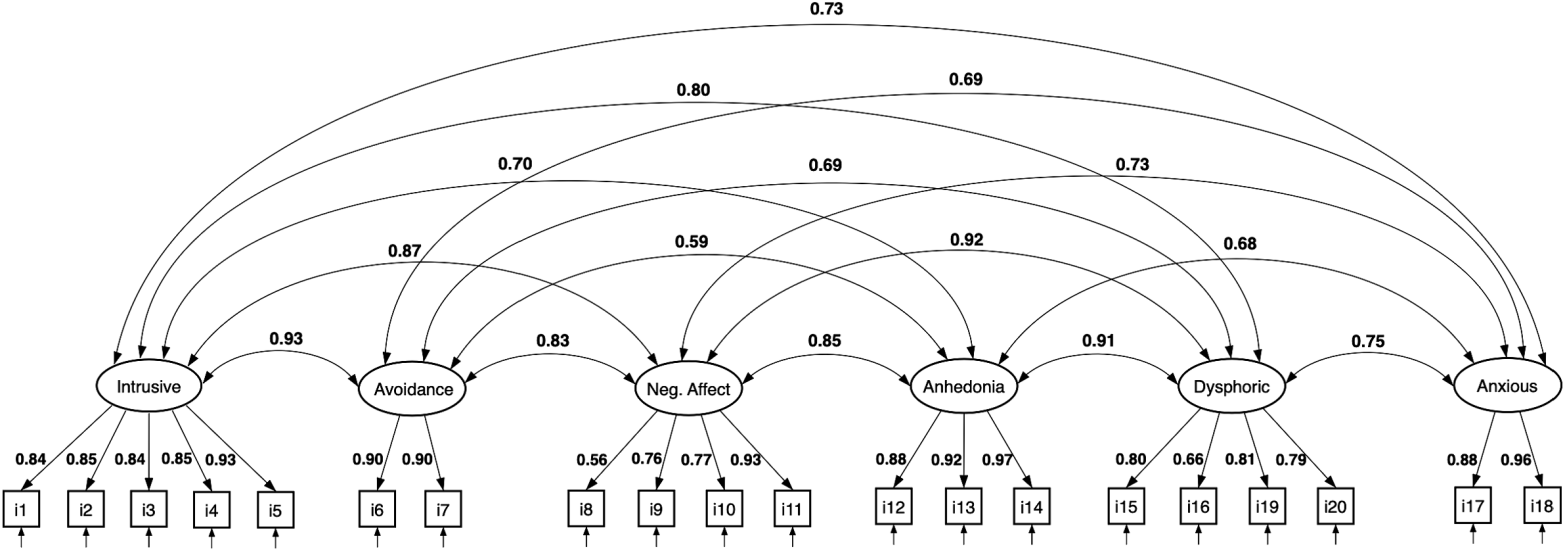
CFA of the six-factor anhedonia model. All estimates are standardized.

**Figure 4:**
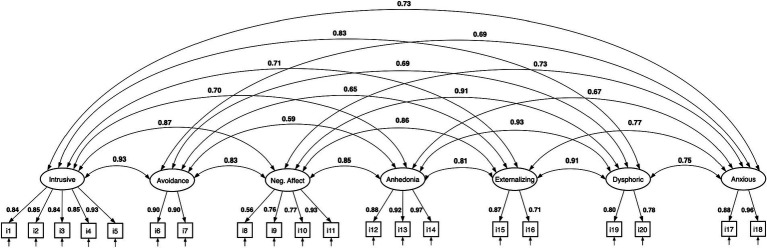
CFA of the seven-factor hybrid model. All estimates are standardized.

Figures 2–4 show the structure of the models, the standardized factor loadings, and the correlations between the latent factors. The four-factor model only had a partial fit (RMSEA = 0.071, RMSEA 95% CI 0.061–0.080, CFI = 0.993, TLI = 0.992, SRMR = 0.069) because the RMSEA 95% confidence interval was above the threshold and its chi-square test was unsatisfactory ( $\chi^{2}$ = 370.37, *p* < 0.001). However, the other indices (CFI, TLI, and SRMR) were satisfactory. A summary of the CFA analyses is shown in Table 5.

**Table 5:** Summary of the CFA for the DSM-5, anhedonia, and hybrid PTSD models.

	$\chi^{2}$ (df)	RMSEA	RMSEA 95% CI	CFI	TLI	SRMR	*p*
DSM-5	370.37 (164)	0.071	0.061–0.080	0.993	0.992	0.069	<0.001
Anhedonia	157.81 (155)	0.008	0.000–0.031	1.000	1.000	0.050	0.422
Hybrid	151.82 (149)	0.009	0.000–0.031	1.000	1.000	0.048	0.420

Following the criteria we considered for evaluating the CFA models, the anhedonia model ( $\chi^{2}$ = 157.81, *p* = 0.422) and the hybrid model ( $\chi^{2}$ = 151.82, *p* = 0.42) showed a better fit than the DSM-5 four-factor model and met all of the criteria we considered for an acceptable model (i.e., CFI and TLI > 0.95, RMSEA <0.06, SRMR <0.08, Hu and Bentler, 1999). The scaled chi-square difference test (Satorra and Bentler, 2010) showed that, compared to the four-factor DSM-5 model, both the anhedonia [ $\chi^{2}$ *diff* (9) = 67.86, *p* < 0.001] and the hybrid model [ $\chi^{2}$ *diff* (15) = 89.16, *p* < 0.001] provided a significantly better model fit. However, when the anhedonia and hybrid models were compared with one another, the chi-square difference test yielded nonsignificant results, indicating that the anhedonia and the hybrid models did not outperform one another [ $\chi^{2}$ *diff* (6) = 4.19, *p* = 0.138].

## Discussion

In the present study, we systematically translated the PCL-5 from English to Spanish, validated it linguistically and assessed its psychometric properties in a sample of individuals 6 months after having a traumatic brain injury. To our knowledge, this was the first study to thoroughly validate the Spanish version of the PCL-5. The participants’ mean score on the PCL-5 was low, and the distribution was right skewed. Only 12.2% of the TBI patients had elevated PTSD scores at 6 months after the injury, which is comparable to previous findings from civilians (Bryant et al., 2001; Bombardier et al., 2006; Haarbauer-Krupa et al., 2017). Furthermore, the association between sex and PTSD incidence was not significant, despite previous results indicating a greater PTSD risk for women after TBI (Olff et al., 2007). Overall, the Spanish translation of the PCL-5 showed excellent internal consistency. The Cronbach’s alpha coefficient of 0.94 that was obtained herein is similar to the Cronbach’s alpha reported for the English version as well as for the German and French translations of the PCL-5 (Blevins et al., 2015; Ashbaugh et al., 2016; Krüger-Gottschalk et al., 2017). The McDonald’s omega coefficient and the split-halve reliability values also confirmed the reliability of the Spanish translation of the PCL-5 instrument. Similarly, the item-total correlations were satisfactory, and only two items showed relatively lower correlations— item 8 “Dissociative amnesia” and item 16 “Recklessness and self-destruction.” Interestingly, item 16, which had the lowest item-total correlations among all the items, is one of the newly symptoms in the DSM-5.

As expected, strong correlations between PTSD, GAD, and depression were detected, which is also in line with the literature and confirms the concurrent validity of the Spanish translation of the PCL-5 instrument. For example, using the same instruments as in the present study, Bovin et al. (2016) reported a correlation of 0.74 between the PHQ-9 and the PCL-5 instruments. Durham et al. (2015) also reported similar correlations between GAD-7 scores and PTSD symptoms, ranging from 0.5 to 0.6. Another study on US combat soldiers, however, reported stronger correlations (ranging from 0.78 to 0.79) between the GAD-7 score and the PCL-5 score (Hoge et al., 2014). These results were expected because PTSD highly overlaps with depression and GAD, and distinguishing it from mood and other anxiety-related disorders has been a challenge for clinicians (Yehuda and McFarlane, 1995). For example, depression frequently cooccurs with PTSD and is estimated to be present in 30–50% of PTSD patients (Nixon et al., 2004; Campbell et al., 2007). Keane et al. (1997) reported four reasons for the comorbidity of these disorders: (1) the PTSD construct shares multiple symptoms with depression and GAD, (2) PTSD is more likely to appear in individuals vulnerable to stress, (3) PTSD might increase the probability of other mental disorders over time, and (4) PTSD frequently appears with other disorders, particularly GAD and major depressive disorder (MDD) (Brown et al., 2001; Post et al., 2011; Contractor et al., 2015).

However, despite the comorbidity of PTSD, depression, and GAD, the results of the exploratory factor analysis indicated that the items of these instruments load on distinct factors, which further supports the validity of the PCL-5 instrument. Interestingly, however, items of the PCL-5 and PHQ-9 assessing disordered eating and sleep disturbance, loaded only on the GAD-7 instrument; however, their loading was the lowest among the items of this factor, suggesting that disordered eating and sleep disturbance could form different factors if they were accompanied by other items reflecting these two outcomes, which should be examined in future studies.

We also examined the structural validity of the PCL-5 using three PTSD models. The CFA analyses showed that the PTSD model derived from the DSM-5 diagnostic categories partially met the specified criteria for a valid model, in contrast to the anhedonia and the hybrid models, which showed adequate and significantly better fits than did the DSM-5 model. The analysis also indicated that the difference between the anhedonia and hybrid models was not significant. However, both the anhedonia and hybrid models had a CFI and TLI of 1.00, which indicates overfitting of the model. Although these models provide better goodness of fit than the four-factor model, an overfitted model limits the generalization of the results and hints that a simpler model is favorable; i.e., the additional complexity of the six- and seven-factor models is not desirable. The high correlation between the anhedonia and the hybrid models indicated that the suggested factors might be redundant and that the improved model fit might be an artifact of the additional complexity as well as the greater correlation between the factors. The strong correlation between the factors also explains why parallel analysis suggested 2 factors for the PCL-5 instrument. In addition to the concerns regarding the overfitting of the anhedonia and the hybrid models, the results of the chi-square statistics must also be interpreted with caution (Bryant and Satorra, 2012). This test is known to be liberal, even with the WLSMV estimator (Muthén et al., 1997). For example, the WLSMV estimator is known to perform well for sample sizes as small as 200; however, a sample size of 400 is recommended for skewed distributions (Muthén et al., 1997; Liang and Yang, 2014).

Evaluating factorial structures solely based on fitness indices is not recommended, and the theoretical foundation and implications of the models should be taken into consideration, especially when presumptuous statistical procedures such as confirmatory factor analysis are utilized. For example, the DSM-5 model is formulated using the diagnostic categories and criteria of the DSM-5. These categories facilitate PTSD diagnosis by grouping conceptually distinct symptoms that are not mutually exclusive for a PTSD diagnosis. For the diagnosis of PTSD, individuals with negative cognitive or mood symptoms or a combination of both would meet the criteria of category D. Although it is known that cognition and emotions influence one another (Pessoa, 2008), there is a large body of literature to conclude that they involve different processes that justify their differentiation (Power and Dalgleish, 2015). Therefore, grouping negative cognitive and mood symptoms according to diagnostic criteria is practical because of the simplification of diagnostic criteria or because these symptoms are comorbid and not mutually exclusive. Therefore, although the results of the CFA analysis support the structural validity of the instrument, it is inconclusive which model is more trustworthy or applicable. There is clearly a need for further research to better understand symptoms of PTSD and its factorial structure.

The current study has a few limitations. First, the data used in the study came from a larger observational study; thus, data were not collected for validating the Spanish translation of the PCL-5 instrument. As a result, some of the typical methods for examining an instrument, such as test–retest reliability, were not performed. For the same reason, the compatibility of the Spanish translation of the PCL-5 with structured clinical interviews was not assessed; thus, it is not clear to what extent the provisional diagnoses we are reporting are accurate or comparable with rates reported in the literature. In addition, our data suffered from a floor effect, with most of the subjects scoring very low on the PCL-5 instrument. To address this problem, however, we applied the WLSMV estimator instead of the maximum likelihood estimator, which is also more suitable for ordinal data (Beauducel and Herzberg, 2006). Finally, the incidence of PTSD is known to vary considerably in different countries (Burri and Maercker, 2014); thus, the reader should consider that the data of the current study were collected solely from four cities in Spain and only from adults with traumatic brain injuries.

## Conclusion

Overall, our results suggest that the Spanish translation of the PCL-5 has good internal consistency and reliability and, similar to the English, French, and German versions of the instrument. The instrument’s validity was also confirmed, both with EFA and CFA analyses as well as correlation with the PHQ-9 and GAD-7 instruments. Future studies should further examine sex differences in the response to different diagnostic categories of PTSD. In addition, the relationships of disordered eating and sleep disturbances with PTSD and GAD should be further investigated.

## Data availability statement

The data of the analysis are not published in the repository to protect sensitive information about the participants. However, access to the data can be requested from CENTER-TBI via https://center-tbi.eu/data.

## Ethics statement

This study was approved by the CENTER-TBI ethical committee. The CENTER-TBI study (EC grant 602,150) was conducted in accordance with all relevant laws of the EU if directly applicable or because of direct effects and all relevant laws of the country where the recruiting sites were located, including but not limited to the relevant privacy and data protection laws and regulations (the “Privacy Law”), the relevant laws and regulations on the use of human materials, and all relevant guidance relating to clinical studies from time to time in force, including but not limited to the ICH Harmonized Tripartite Guideline for Good Clinical Practice (CPMP/ICH/135/95) (“ICH GCP”) and the World Medical Association Declaration of Helsinki titled “Ethical Principles for Medical Research Involving Human Subjects.” Informed consent was obtained from the patients and/or the legal representative/next of kin, according to the local legislation, for all patients recruited in the Core Dataset of CENTER-TBI and documented in the e-CRF. Ethical approval was obtained for each recruiting site. The list of sites, ethical committees, approval numbers and approval dates can be found on the following website: https://www.center-tbi.eu/project/ethical-approval.

## Author contributions

EFH conceived, planned, and performed the analysis and wrote the manuscript. JS, AR, IP, and GL collected the data. All authors contributed to the article and approved the submitted version.

## Footnotes

^1^www.incf.org

## Appendix

**Spanish translation of the posttraumatic stress disorder checklist (PCL-5)** Instrucción: Más abajo hay un listado de problemas que las personas tienen a veces debido a una experiencia muy estresante. Lea por favor cada problema detenidamente y después rodee uno de los números de la derecha para indicar con cuánta intensidad le ha molestado aquel problema durante el último mes.

**Table:**

En el último mes, ¿cuánto le ha molestado		Nada	Un poco	Moderadamente	Bastante	Muchísimo
1	Tener recuerdos repetidos, perturbadores y no deseados de la experiencia estresante?	0	1	2	3	4
2	Tener sueños repetidos, perturbadores de la experiencia estresante?	0	1	2	3	4
3	Sentirse o actuar de repente como si la experiencia estresante volviera a suceder (como si realmente estuviera allí reviviéndolo)?	0	1	2	3	4
4	Sentirse muy disgustado cuando algo le recordaba la experiencia estresante?	0	1	2	3	4
5	Tener reacciones físicas intensas cuando algo le recordaba la experiencia estresante (p.ej. palpitaciones, dificultades para respirar, sudoración)?	0	1	2	3	4
6	Evitar recuerdos, pensamientos o sentimientos relacionados con la experiencia estresante?	0	1	2	3	4
7	Evitar estímulos externos relacionados con la experiencia estresante (p.ej. personas, lugares, conversaciones, actividades, objetos o situaciones)?	0	1	2	3	4
8	Tener dificultades para recordar partes importantes de la experiencia estresante?	0	1	2	3	4
9	Tener fuertes creencias negativas sobre uno mismo, otras personas, o el mundo (p.ej. tener pensamientos como: no soy una buena persona, hay algo que seriamente no está bien en mí, no se puede confiar en nadie, el mundo es muy peligroso)?	0	1	2	3	4
10	Culparse a sí mismo o a otra persona dela experiencia estresante o de lo que sucedió después?	0	1	2	3	4
11	Tener fuertes sentimientos negativos tales como miedo, terror, ira, culpa o vergüenza?	0	1	2	3	4
12	Perder el interés en actividades que antes solía disfrutar?	0	1	2	3	4
13	Sentirse distante o apartado de los, demás?	0	1	2	3	4
14	Dificultades para sentir emociones positivas (p.ej. ser incapaz de sentir alegría o tener sentimientos de amor hacia personas cercanas)?	0	1	2	3	4
15	Tener conductas irritables, ataques de ira o actuar de manera agresiva?	0	1	2	3	4
16	Tomar demasiados riesgos o hacer cosas que Ie pudieran dañar?	0	1	2	3	4
17	Estar muy en alerta o en guardia?	0	1	2	3	4
18	Sentirse asustadizo o sobresaltado?	0	1	2	3	4
19	Tener dificultades para concentrarse?	0	1	2	3	4
20	Tener problemas para dormir o mantener el sueno?	0	1	2	3	4
